# Design and Implementation of a Real-Time Multi-Beam Sonar System Based on FPGA and DSP

**DOI:** 10.3390/s21041425

**Published:** 2021-02-18

**Authors:** Haowen Tian, Shixu Guo, Peng Zhao, Minyu Gong, Chao Shen

**Affiliations:** Key Laboratory of Acoustics Research, China Jiliang University, Hangzhou 310018, China; bryan_thw@163.com (H.T.); zhaopeng@cjlu.edu.cn (P.Z.); ingminyu@163.com (M.G.); chaoshen@cjlu.edu.cn (C.S.)

**Keywords:** 3-D positioning multibeam sonar, real-time system, array signal processing, hardware implementation, multicore pipeline architecture, beamforming algorithm, FPGA, DSP

## Abstract

Aiming at addressing the contradiction between the high-speed real-time positioning and multi-channel signal processing in multi-beam sonar systems, in this work we present a real-time multi-beam sonar system based on a Field Programmable Gate Array (FPGA) and Digital Signal Processing (DSP) from two perspectives, i.e., hardware implementation and software optimization. In terms of hardware, an efficient high-voltage pulse transmitting module and a multi-channel data acquisition module with time versus gain (TVG) compensation with characteristics such as low noise and high phase amplitude consistency, are proposed. In terms of algorithms, we study three beamforming methods, namely delay-and-sum (D&S), direct-method (DM) and Chirp Zeta Transform (CZT). We compare the computational efficiency of DM and CZT in the digital domain. In terms of software, according to the transmission bandwidth of the Gigabit Ethernet and a serial rapid IO (SRIO) interface, the data transmission paths of the acquired data and the beam pattern between the FPGA, the DSP, and a personal computer (PC) are planned. A master-slave multi-core pipelined signal processing architecture is designed based on DSP, which enhances the data throughput of the signal processor by seven times as compared with that of the single-core operation. The experimental results reveal that the sound source level of the transmitting module is around 190.25 dB, the transmitting beam width is 64° × 64°, the background noise of the acquisition module is less than 4 μVrms, the amplitude consistency error of each channel is less than −6.55 dB, and the phase consistency error is less than 0.2°. It is noteworthy that the beam number of the sonar system is 90 × 90, the scanning angle interval is 0.33°, the working distance ranges from 5 m to 40 m, and the maximum distance resolution is 0.384 m. In the positioning experiment performed in this work; the 3-D real-time position of the baffle placed in the detection sector is realized. Please note that the maximum deviation of azimuth is 2°, the maximum deviation of elevation is 2.3°, and the maximum distance deviation is 0.379 m.

## 1. Introduction

The design of modern sonar systems is generally based on the use of a hydrophone array for improving the ability of underwater target location and recognition [1,2]. In the sonar system, the technical means of using the relative delay of the array signal for in-phase superposition to achieve the target location is called beamforming. Currently, the development of high-speed real-time active sonar systems with multi-beams as the core technology is a focus of research community in the field of underwater acoustic engineering [3,4]. In multi-beam sonar systems, the main factor which influences the angular resolution is the aperture of the hydrophone array. Increasing the aperture of the array leads to the narrowing of the main lobe width of the natural beam angle, thus improving the angular resolution of the positioning [5,6], however, this increases the aperture of the array in order to avoid the grating lobe effect, i.e., the hardware needs to provide more signal acquisition channels [7], but it is notable that the volume of the hardware system and its internal signal processing unit are usually limited. Therefore, increasing the aperture not only adds to the burden on the hardware system, but also increases the number of calculations required of the signal processor [8]. Consequently, the system is unable to provide the desired real-time positioning. At the same time, in order to improve the range resolution, the time interval required for processing the frame of echo signal in the multi-beam sonar system is shortened. This phenomenon leads to an increase in the amount of input data per unit time and puts forward higher requirements for the throughput of the signal processor [9]. Therefore, realizing high-speed real-time positioning and high-resolution positioning with limited hardware resources is a complicated task. With the development of underwater acoustic engineering, there is a dire need for real-time performance of multi-beam sonar systems. Therefore, it is of great significance and value to design a multi-beam sonar system with high computational performance that can be used in underwater acoustic engineering applications [10,11].

Recently, a large number of researchers have made important contributions in the design of transmitters, receivers and signal processors. In the study of transmitters, Kazimierczulk analyzed the performance of a class D power amplifier under different switching frequency and load, and obtained the analytical equations of the power amplifier performance parameters [12]. Budihardjo accurately evaluated and verified SPICE models commonly used in power MOSFET circuit design [13]. In order to speed up the switching speed of power MOSFETs and reduce the switching loss, Hwu introduced an efficient low-side gate drive circuit design method [14], and Rosnazri used discrete components to design a high-side high-frequency gate drive circuit [15]. In order to improve the power transfer efficiency of power amplifier circuits, Hurley introduced in detail the design method of push-pull high frequency transformers in switch circuits [16]. Mathams analyzed the signal transmission characteristics of underwater acoustic transducers and introduced a passive network impedance matching method [17]. In the research on receivers, Gao proposed an optimal design method for a wideband preamplifier, which can effectively improve the output signal-to-noise ratio of hydrophones [18]. Jensen studied the characteristics of sound propagation and analyzed the loss of sound propagation in sea, which provided a theoretical basis for the design of a time versus gain (TVG) circuit in the receiver [19]. Aiming at solving the problem of multi-channel data acquisition of large arrays and real-time data interaction between upper and lower computers, Lin designed a data acquisition system with a playback function based on FPGA [20], and Bailey transmited the acquisition data from a FPGA to a PC in real time through Gigabit Ethernet [21]. In the related research on signal processors, Palmese studied a beamforming algorithm based on the CZT in uniform linear arrays and planar arrays [22], and then deduced its analytical formula in near field in detail [23]. Zhan introduced the method of high-speed serial communication between a FPGA and DSP through SRIO [24]. Yang used multiple DSP chips to form a signal processing array to achieve high-precision sonar imaging [25].

However, most sonar systems are limited in size. When the hardware system resources are limited, the contradiction between multi-channel signal processing and high-speed real-time positioning will be more severe. In this work, we propose a design method of a real-time multibeam sonar system with the feature of 90 × 90 beams, 0.384 m distance resolution, 190.25 dB sound source level, and background noise less than 4 μVrms to realize real-time 3-D position of target objects in a 30° × 30° sector at a distance of 5 m to 40 m (for the purpose of explaining the function and performance of the system, this working distance range has been considered sufficient, and it is convenient for the actual performance testing of the system. According to Section 3, the working distance can be changed by hardware design). The intended application of this multi-beam sonar system is ROV navigation, obstacle avoidance, search and recovery and defense and security. Compared with the existing multi-beam sonars, such as the SeaBat 7128, the number of beams generated by the sonar presented in this paper is 32 higher and the real-time target location can be carried out in the 3-D sector. First, we describe the physical working principle and spatial signal processing methods used in the proposed multi-beam sonar system. In addition, we also focus on 2-D CZT beamforming algorithm. Second, we present the proposed hardware architecture which is based on FPGA and DSP. The design methods of a high-voltage pulse transmitter module and multi-channel synchronous acquisition module with wide dynamic range and low noise in the hardware system are also expounded. Third, we present the workflow of the software used in this work. In addition, we also propose a DSP-based master-slave multi-core pipelined signal processor architecture. Moreover, we also discuss the conditions for real-time signal processing under the proposed architecture. Lastly, we test and evaluate the sound source level, received background noise, received phase and amplitude consistency and 3-D positioning performance of the multi-beam sonar system. The experimental results show that the proposed system has the ability to realize the real-time 3-D positioning of underwater targets under the design background requirements. In addition, the scalability of the system is also analyzed.

## 2. Operating Principle of Multibeam Sonars

### 2.1. The Positioning Method of Multibeam Sonars

**Figure 1:**
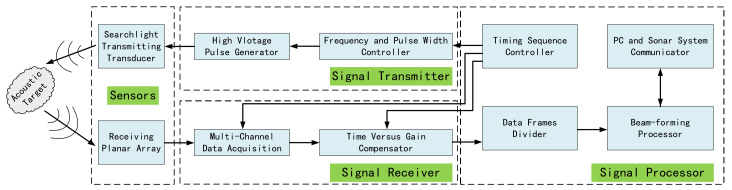
The architecture of multibeam sonar.

Figure 1 presents the overall architecture of the proposed multi-beam sonar system. The multi-beam sonar system is composed of a transducer array, signal transmitter, signal receiver and signal processor. The transmitter generates a high-voltage pulse signal to stimulate the transmitting transducer to project the ultrasonic pulse on the target sector. As the target object is at a particular position in the target sector, the underwater propagation impedance of the ultrasound is not continuous. The hydrophone array is excited by the reflected wave from the target object. After each ultrasonic pulse is transmitted, the receiver performs a certain time continuous synchronous data acquisition and TVG compensation for multiple hydrophone array elements according to the detection distance and divides the continuous received data into several signal frames on the basis of the pulse width of the ultrasonic signal. When a signal frame is received by the signal processor, the corresponding time delay compensation is carried out for each acquisition signal in multiple predetermined directions. Please note that all the echo signals are superimposed and summed to calculate the echo intensity in each direction which is then used to obtain the corresponding beam pattern.

All the beam patterns calculated in a detection cycle are spliced in time, and finally a 3-D distribution of the target object in the detection sector is obtained. In this work, we propose a multi-beam sonar system which uses a wide beam to emit a 100 KHz continuous wave (CW) ultrasonic signal and receives the echo signal of the underwater target through a 2-D uniform planar array. In order to increase the aperture of the array without changing the number of hydrophones, the array elements are arranged by twice the wavelength. As a result, a grating lobe effect is not generated when the detection sector is limited to 30° × 30°. The method of calculating the intensity of echo signals originating from multiple predetermined directions is called beamforming. There are different beamforming techniques which are used to improve the positioning efficiency based on the application at hand.

### 2.2. Study on Beamforming Method for Planar Arrays

**Figure 2:**
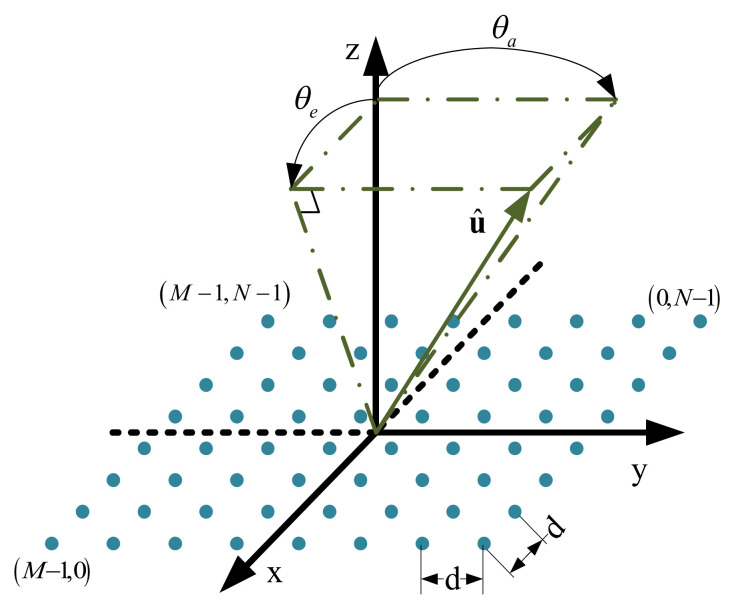
Definition of elevation and azimuth for a 8 × 8 2-D array.

Under real-time working conditions, the number of beams is a key factor to measure the signal processing performance of a multi-beam sonar system. Therefore, in this section, we studied the D&S, DM, and CZT beamforming algorithms based on uniform planar array and the calculation efficiency of DM and CZT under different beam numbers is discussed in detail. For a *M* × *N* uniform plane receiving array which has array elements with spacing *d*, the coordinate indexed as (*m*, *n*) element is expressed as (*md*, *nd*, *0*). According to Fresnel approximation, the delay compensation parameter *τ*(*m*, *n*, *r*_0_) of (*m*, *n*) element is expressed as follows:

(1) $$\tau \left(m,n,r_{0}\right)=\frac{\left(md,nd,0\right)\cdot \hat{u}}{c}-\frac{{\left(md\right)}^{2}+{\left(nd\right)}^{2}}{2r_{.0}c}$$

where, $\hat{u}$ represents the unit direction vector of the array, *c* represents the underwater sound speed, and *r*_0_ represents the distance. $\hat{u}\;$ is expressed as follows:

(2) $$\hat{u}=\left(\sin\theta_{e},\sin\theta_{a},\sqrt{\cos^{2}\theta_{e}-\sin^{2}\theta_{a}}\right)$$

where, *θ_e_* is used to represent the elevation angle, which is defined as the angle between vector $\hat{u}$ and its projection on the YOZ plane. *θ_a_* is used to represent the azimuth angle, which is defined as the angle between vector $\hat{u}$ and its projection on the YOZ plane. Figure 2 intuitively depicts the definition of elevation and azimuth of a 2-D uniform plane array, which is conducive to the subsequent signal processing calculation.

The delay-and-sum (D&S) beamforming method calculates *P* × *Q* time-domain beam signals in the detection sector. The beam signal *b*(*t*, *r*_0_, *θ_e__p_*, *θ_ap_*) is expressed as follows:

(3) $$b(t,r_{0},\theta_{ep},\theta_{aq})=\sum_{m=0}^{M-1}\sum_{n=0}^{N-1}w_{m,n}s_{m,n}\left(t-\tau \left(m,n,r_{0},\theta_{ep},\theta_{aq}\right)\right)$$

where, *r*_0_ represents the distance, *w_m,n_* denotes the array weighting coefficient, *s_m,n_*(*t*) denotes the time domain echo signal received by *M* × *N* array element, and *t* represents the time. Please note that when D&S beamforming method is used for spatial signal processing in digital systems, the sampling rate of the front-end signal of the system directly affects the positioning output.

The beam intensity in digital systems is often represented by the frequency domain mode of D&S beamforming. Assuming that *f_s_* denotes the sampling frequency and *K* denotes the sampling snapshot number, the FFT of beam signal presented in (3) at *K*-points is expressed as follows:

(4) $$\begin{array}{cc} B(k,r_{0},\theta_{ep},\theta_{aq}) & =\left|\sum_{m=0}^{M-1}\sum_{n=0}^{N-1}w_{m,n}S_{m,n}\left(k\right)\exp\left(-j2\pi \frac{k}{K}f_{s}\tau \left(m,n,r_{0},\theta_{ep},\theta_{aq}\right)\right)\right|\\ & =\left|\sum_{m=0}^{M-1}\sum_{n=0}^{N-1}w_{m,n}S_{m,n}\left(k\right)\varphi \left(k,m,n,r_{0},\theta_{ep},\theta_{aq}\right)\right| \end{array}$$

where, *S_m,n_*(*k*) denotes the spectral coefficient of *s_m,n_*(*t*), and *k* denotes the index of the frequency spectral line.

Equation (4) is a general expression that represents direct-method (DM) beamforming in the frequency domain. When using DM beamforming to calculate each *B*(*k*, *r*_0_, *θ_ep_*, *θ_ap_*), the digital system completes the initialization of a *φ*(*k*, *m*, *n*, *r*_0_, *θ_ep_*, *θ_ap_*) in advance and saves it in the cache as a lookup table. As compared with the time domain processing, although it is no longer the main factor affecting the positioning effect, because the calculation of DM beamforming method mainly concentrates on the *M* × *N* × *P* × *Q* complex multiplication. So, the digital system needs to adopt four-layer nested loop for computing (4). This method is not competent enough in a system with a high refresh rate of positioning results.

Under the premise that the elevation angle *θ_e_* and azimuth *θ_a_* of the detection sector are equal interval values, the accelerated calculation method of (4) is proposed in [22] and [23]. The (4) can now be expressed in terms of the following complex polynomial:

(5) $$B(k,\theta_{ep},\theta_{aq})=\left|W_{e}^{\frac{-p^{2}}{2}}W_{a}^{\frac{-q^{2}}{2}}\sum_{m=0}^{M-1}\left(\sum_{n=0}^{N-1}v_{m,n}S_{m,n}\left(k\right)A_{a}^{n}W_{a}^{\frac{-n^{2}}{2}}W_{a}^{\frac{{\left(q-n\right)}^{2}}{2}}\right)A_{e}^{m}W_{e}^{\frac{-m^{2}}{2}}W_{e}^{\frac{{\left(p-m\right)}^{2}}{2}}\right|$$

where, *v_m,n_* is defined as follows:

(6) $$v_{m,n}=w_{m,n}\exp\left(j2\pi \frac{k}{K}f_{s}\left({\left(m-\frac{M-1}{2}\right)}^{2}+{\left(n-\frac{N-1}{2}\right)}^{2}\right)\frac{d^{2}}{2r_{0}c}\right)$$

and *A_e_*, *W_e_*, *A_a_*, *W_a_* are defined as follows:

(7) $$\left\{\begin{array}{l} A_{e}=\exp(-j2\pi \frac{k}{K}f_{s}\frac{d\sin\theta_{ei}}{c})\\ W_{e}=\exp(j2\pi \frac{k}{K}f_{s}\Delta s_{e})\\ \Delta s_{e}=\frac{\sin\theta_{ef}-\sin\theta_{ei}}{P-1} \end{array}\right.\left\{\begin{array}{l} A_{a}=\exp(-j2\pi \frac{k}{K}f_{s}\frac{d\sin\theta_{ai}}{c})\\ W_{a}=\exp(j2\pi \frac{k}{K}f_{s}\Delta s_{a})\\ \Delta s_{a}=\frac{\sin\theta_{af}-\sin\theta_{ai}}{Q-1} \end{array}\right.$$

where, *θ_ei_* and *θ_ai_* represent the scanning start points of elevation and azimuth, respectively and *θ_ef_* and *θ_af_* represent the scanning endpoints of elevation and azimuth, respectively. In addition, it is necessary that $\left|\theta_{ei}\right|,\left|\theta_{ai}\right|,\left|\theta_{ef}\right|,\left|\theta_{af}\right|\leq \pi /2$, and *P* × *Q* are the total number of beam signals in frequency domain, and Δ*s_c_* represents the sine value of the elevation angle resolution, and Δ*s_a_* represents the sine value of the azimuth angle resolution. When k is given, Equation (5) is expressed by the two-dimensional discrete linear convolution of **C***_M,N_* and **D***_P+M−_*_1*,Q+N−*1_ matrices as presented in Equation (8):

(8) $$\left\{\begin{array}{l} C_{M,N}=v_{m,n}S_{m,n}\left(k\right)A_{e}^{m}W_{e}^{\frac{-m^{2}}{2}}W_{e}^{\frac{{\left(p-m\right)}^{2}}{2}}A_{a}^{n}W_{a}^{\frac{-n^{2}}{2}}W_{a}^{\frac{{\left(q-n\right)}^{2}}{2}}\\ D_{P+M-1,Q+N-1}=W_{e}^{\frac{p^{2}}{2}}W_{a}^{\frac{q^{2}}{2}} \end{array}\right.$$

where, *m = 0*, *…, M* − 1, *n* = 0, *…*, *N* − 1 and *p* = 0, *…*, *P + M* − 1, and *q =* 0, *…*, *Q + N* − 1. According to the relationship between the linear convolution and circular convolution, *L*_1_ ≥ *P* + 2*M* − 2, *L*_2_ ≥ *Q* + 2*N* − 2, and *L*_1_, *L*_2_ = 2*^N^*^+^ needs to be satisfied, where *N+* denotes the set of positive integers. According to the convolution theorem, the spatial beam intensity matrix **B***_P,Q_* is expressed as follows:

(9) $$B_{P,Q}=\left|{\left[IFFT2_{L_{1},L_{2}}\left[FFT2_{L_{1},L_{2}}\left[C_{M,N}\right]\;\times \;FFT2_{L_{1},L_{2}}\left[D_{P+M-1,Q+N-1}\right]\right]\right]}_{P,Q}\right|$$

where, $FFT2_{L_{1},L_{2}}\left[\;\right]$ means that the matrix in parentheses is zero-padded to attain a size equal to *L*_1_ × *L*_2_. $IFFT2_{L_{1},L_{2}}\left[\;\right]$ means that the matrix in parentheses is transformed by 2-D IFFT of *L*_1_ × *L*_2_ size. [ ]*_P,Q_* means that the matrix in parentheses is truncated by *P* × *Q* size. When the digital system performs CZT beamforming, the coefficients of *S_m,n_*(*k*) in **C***_M,N_* matrix are calculated at system’s initialization, and the 2-D FFT transform after zero padding of **D***_P+M−1,Q+N−1_* matrix is also completed. Therefore, the calculation of **B***_P,Q_* is done by the complex multiplication, i.e., *M* × *N* + *L*_1_ × *L*_2_ × *log*_2_*L*_1_ + *L*_1_ × *L*_2_ × *log*_2_*L*_2_ + *L*_1_ × *L*_2_.

**Figure 3:**
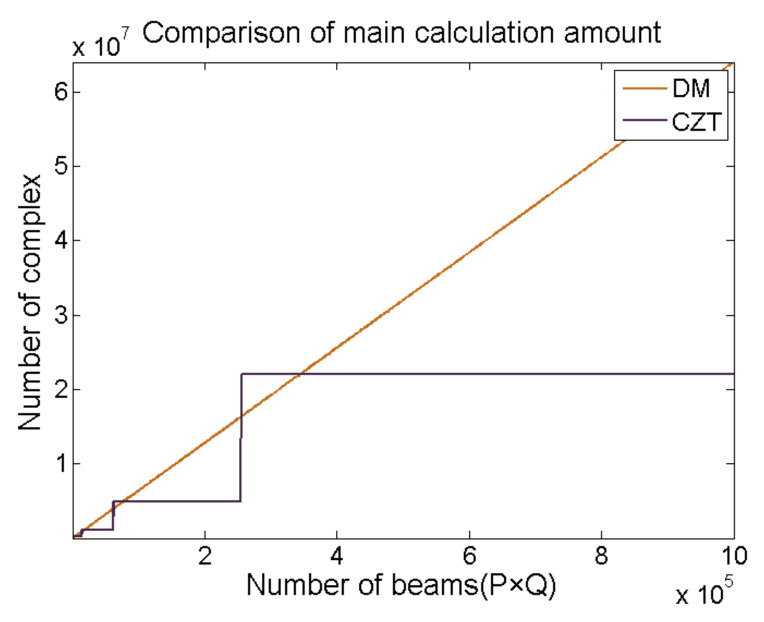
Comparison of main calculation amount.

Although [22,23] compared the complexity of D&S, DM and CZT beamforming algorithms at the analytical level, and obtained the conclusion that the performance of CZT is always the optimal, according to Equations (3)–(5), some parameters of the three beamforming algorithms in the digital system can be initialized, and we found that the computational efficiency of DM was not completely lower than that of CZT. In the analytic formula, both D&S and DM are calculated in the form of 2-D multiplication and addition, but D&S is used to calculate beam signals with a time series *s_m,n_*(*t*), because k is fixed and DM is used to calculate beam signals with a complex parameter *S_m,n_*(*k*). Thus, although the physical meaning of D&S is very obvious, when *K* is equal to 256, the computational efficiency of D&S will be lower than DM thoroughly. Therefore, we only discuss the computational efficiency of DM and CZT methods. Figure 3 compares the computational complexity of the digital signal processing system for achieving DM and CZT in various beam dimensions when *M* = *N* = 8. When *P* + 2*M* − 2 is less than *L*_1_ and *Q* + 2*N* − 2 is less than *L*_2_, **C***_M,N_* and **D***_P+M−1,Q+N−1_* will be zero-padded to the size of *L*_1_ × *L*_2,_ so we can find in Figure 3 that the calculation amount of CZT algorithm does not change with the increase of beam number in a certain region. We can also find that the computational efficiency of CZT is inferior to that of DM when *P* + 2*M* − 2 is slightly larger than a certain 2*^N^*^+^. In this work, because of *P* = *Q* = 90, thus, we can conclude that the CZT algorithm is the best choice.

## 3. Hardware Design

### 3.1. Overall Hardware Design

**Figure 4:**
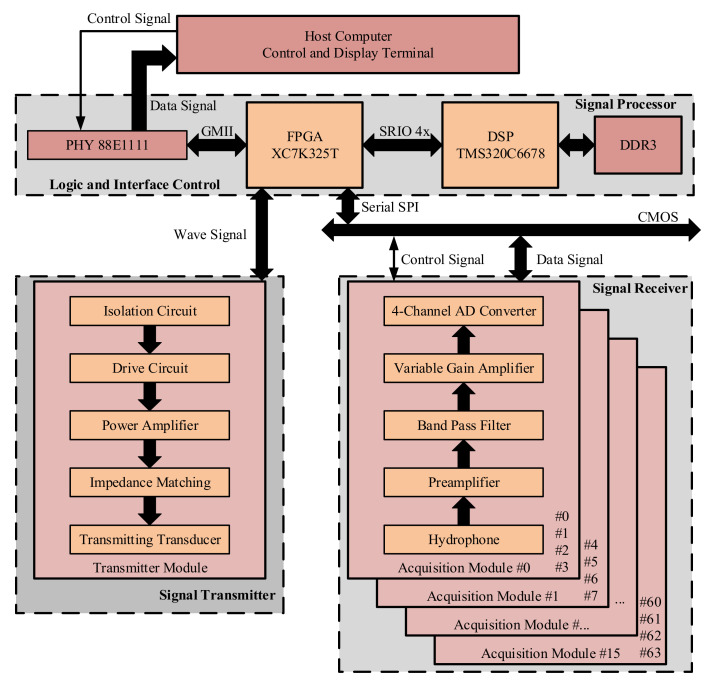
System hardware block diagram.

Figure 4 shows the overall hardware architecture of the proposed multi-beam sonar system. The hardware system is composed of one transmitter module, 16 acquisition modules and the corresponding logic control and signal processing modules. Among them, the transmitter module and the acquisition module are the major design complexities of the sonar hardware system. The logic control and signal processing module composed of FPGA and DSP is the key to implement high-performance 3-D positioning. First, the transmitting module is electrically isolated from the power supply and the logic drive signal of FPGA. This effectively prevents any interference of high-power signal with the receiver and signal processor. At the same time, the MOSFET drive circuit is used to drive the power amplifier. The impedance matching with the transmitting transducer is carried out to realize the high acoustic source level ultrasonic emission.

Second, the acquisition module uses the preamplifier to amplify the output signal of the hydrophone. In addition, it also uses the high-order analog band-pass filter to improve the signal-to-noise ratio. Then, on the basis of VGA, the acquisition module changes the secondary magnification according to the TVG gain curve specified in the FPGA. ADC in each acquisition module synchronously collects four analog signals processed by the conditioning circuit and FPGA synchronizes each ADC through synchronous sampling pulses and reads the collected data through serial SPI. Finally, a XC7K325T Kintex-7 series FPGA, which has 32,6080 logic units, 16,020 KB Block RAM, 10 PLLs, 500 single-ended IOs and 16 gigabyte-transceiver-X (GTX) high-speed serial transceivers [26]. The DSP is a TMS320C6678 C6000 series unit, which has eight cores and supports fixed-point and floating-point operations. The operation ability of each core reaches up to 40 GMACS and 20 GFLOPS. At the same time, each core has 4 MB multi-core shared memory (MSM), 32 KB level 1 program memory (L1P), 32 KB level 1 data memory (L1D), and 512 KB level 2 memory (L2), and L1P, L1D and L2 can be configured as cache or SRAM. The SRIO controller is integrated on the chip. The out-of-chip DDR3 can be extended to 8 GB which supports 1600 MT/s operations. When the system is working, the FPGA controls the Gigabit Ethernet chip 88E1111 through GMII and realizes the high-speed data interaction with the host computer. At the same time, the GTX transceivers are used to connect the SRIO hardware interface at the DSP. The high-speed full duplex serial communication of 5 GBaud per channel is realized [27].

### 3.2. Design of High Voltage Pulse Transmitting Moudle

**Figure 5:**
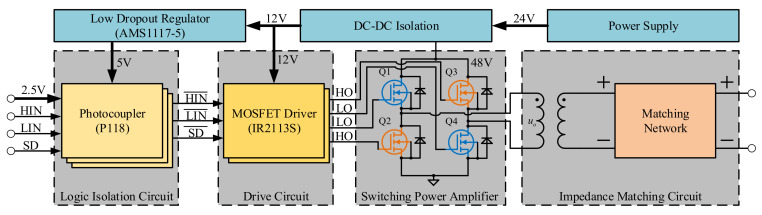
Transmitting module hardware block diagram.

It is necessary for the transmitting module to achieve CW pulse generation at 190 dB sound source level and 100 KHz frequency. As shown in Figure 5, the high voltage pulse transmitting module is mainly composed of a DC-DC isolation power supply, photoelectric coupler, MOSFET driver, switching power amplifier, and impedance matching circuit. As the transmitter module produces continuous high-power pulse signal during its operations, the DC-DC isolation power supply of 12 V and 48 V is used to realize the electrical isolation with the total power supply.

The photoelectric coupler of P118 is used to realize the electrical isolation with the logic control system. (10) denotes the relationship between the sound source level *SL* of the sonar system and the electrical power *P_e_*:

(10) $$SL=170.7+10\mathrm{lg}P_{e}+10\mathrm{lg}\eta +DI_{T}$$

where, *η* represents the efficiency of converting electrical power to acoustic power of transmitting transducer with a value of 50%, *DI_T_* represents the directional gain of emission with a value of 3 dB. Thus, we calculate the value of *P_e_* = 86 W. Considering the efficiency of the transformer and the energy loss of MOSFET, the actual required power is at least 1.5 *P_e_*. In order to improve the efficiency of the power amplification, the transmitting module adopts class D power amplifier and outputs the alternating square wave signal through full bridge inverter circuit [28] composed of power MOSFET Q1, Q2, Q3, and Q4. The MOSFET turn-on voltage drop *V_DS_* is usually equal to 2 V. When the supply voltage *V_DD_* a is 48 V, the maximum current *I_D_*_max_ through MOSFET is expressed as follows:

(11) $$I_{D\max}=\frac{1.5P_{e}}{V_{DD}-2V_{DS}}$$

Now, we calculate the value of *I_D_*_max_ = 3 A. According to the aforementioned analysis, we select the D444 power MOS transistor. The maximum drain-source breakdown voltage of D444 is 60 V, the gate threshold voltage *V_GS(th)_* is 2.4 V, the gate charge *Q_g_* is 7.5 nC, the static drain-source on-resistance *R_DS(on)_* is 47 mΩ, and the on-state drain current *I_D(on)_* is 15 A.

In order to reduce the switching loss of power in MOSFET and further improve the switching speed, we design the MOSFET driving circuit. When the working frequency of the transmitter is 100 KHz, the turn-on time of MOSFET is 5 μs. The gate current provided by the drive circuit should meet the formula as follows:

(12) $$I_{s}>\frac{Q_{g}}{5\;\mu s}=\frac{7.5\mathrm{nC}}{5\;\mu s}=1.5\mathrm{mA}$$

**Figure 6:**
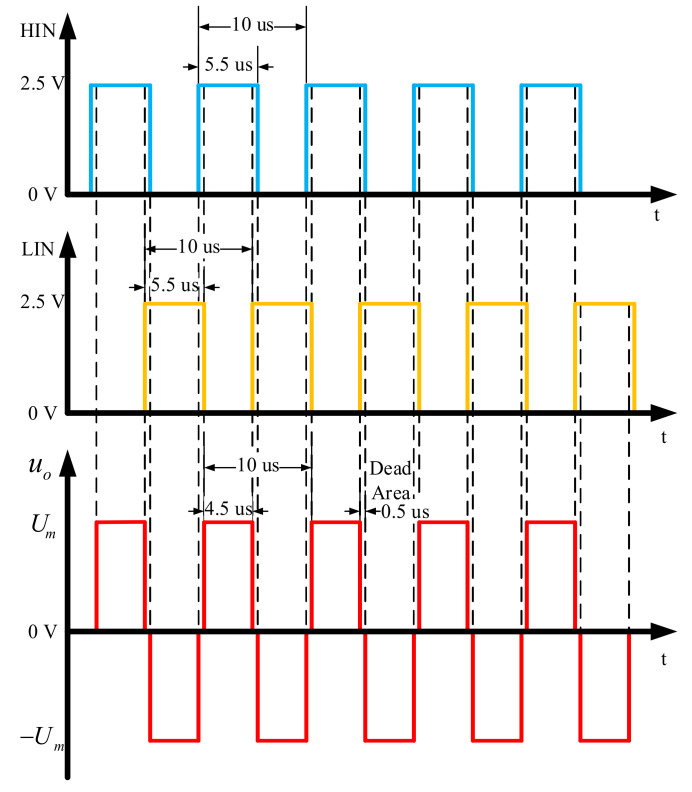
The control timing sequence of CW pulse generator.

We select two IR2113S driver chips in the design of our drive circuit. The low voltage side of IR2113S is compatible with CMOS and TTL levels, the working voltage of high voltage side reaches 500 V, the maximum working current is 4 A, and the working frequency reaches 500 KHz. Through the control of HIN, LIN, SD logic signal, the drive circuit with power amplifier circuit produces 100 KHz high voltage alternating square wave. Figure 6 shows the relationship between the output waveform *u*_0_ of the power amplifier circuit and HIN, LIN, when the operating frequency is 100 KHz and the SD is logic high. *U_m_* represents the maximum output amplitude. The 0.5 μs dead time ensures that the four MOSFETs do not turn on at the same time. By changing the low-level duty cycle of the input logic signal we can realize the power adjustment. Since the ultrasonic transducer is a capacitive load, when *u_0_* passes through the transducer with a resonant frequency of 100 KHz, the high-order harmonic attenuates. Finally, the acoustic signal in the form of 100 KHz sine wave is obtained.

**Figure 7:**
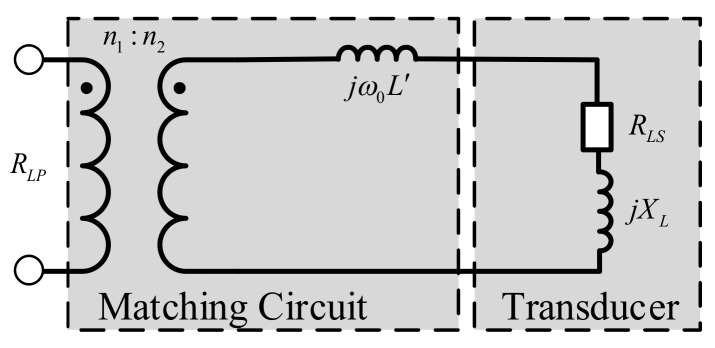
Matching network.

In order to improve the active power obtained by the ultrasonic transducer, it is necessary to design the impedance matching circuit between the output of the power amplifier circuit and the input of the transducer. Figure 7 presents the impedance matching network of the transmitter. Please note that the transmitter module uses a transformer and a series inductance to form a single-peak tuning matching circuit.

The calculation method of transformer primary resistance *R_LP_* is as follows:

(13) $$R_{LP}=\frac{{\left(V_{DD}-2V_{DS}\right)}^{2}}{2P_{T}}$$

where, *P_T_* = 130 W and the resulting value of *R_LP_* = 7.45 Ω. Through the impedance analyzer, we estimate that the conductivity *G_L_* of the transmitting transducer at 100 KHz is 32.78 μS, and the admittance *B_L_* is 92.90 μS. Then, the secondary resistance *R_LS_* of the transformer is expressed as:

(14) $$R_{LS}=\frac{G_{L}}{G_{L}^{2}+B_{L}^{2}}$$

The resultant value of *R_LS_* is 3377.66 Ω. Now, the turns ratio *n*_2_:*n*_1_ of transformer is 23. Finally, the series inductance $L^{\prime }$ of the matching circuit can be expressed as:

(15) $$L^{\prime }=\frac{1}{\omega_{0}}\frac{B_{L}}{G_{L}^{2}+B_{L}^{2}}$$

We can then calculate that *L’* is 15.24 mH.

### 3.3. Design of Multi-Channel Data Acquisition Module

**Figure 8:**
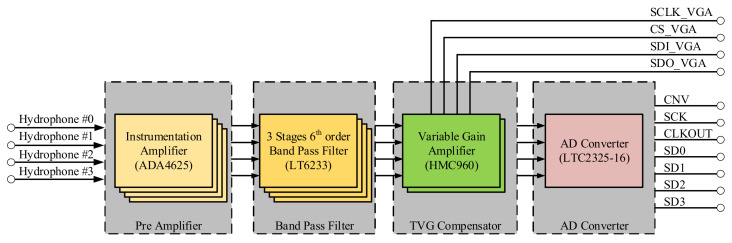
Acquisition module hardware block diagram.

The design of acquisition module is based on four channels. Figure 8 shows the hardware structure of the acquisition module. This module comprises preamplifier, band-pass filter, variable gain amplifier, and analog-to-digital converter. The operating frequency *f*_0_ of the sonar system is 100 KHz, the receiving sensitivity *S_R_* of the planar piston hydrophone is −180 dB, the maximum operating distance *D*_max_ is 40 m, the minimum operating distance *D*_min_ is 5 m, the emission sound source level *SL* is 190 dB, and the receiving directional gain *DI_R_* is 3 dB.

We set the sampling number *N_ad_* of ADC at 16 bit, the reference voltage *V_ref_* at 4.096 V, and the target intensity $TS\in \left(-15\;\mathrm{dB},\;25\;\mathrm{dB}\right)$. By using the spherical wave attenuation model, the relationship between dynamic range of TVG, operating frequency and operating range of sonar system is presented in Equation (16):

(16) $$TVG_{range}=2\left(20\mathrm{lg}\frac{D_{\max}}{D_{\min}}+0.036f_{0}^{1.5}\left(D_{\max}-D_{\min}\right)10^{-3}\right)$$

**Figure 9:**
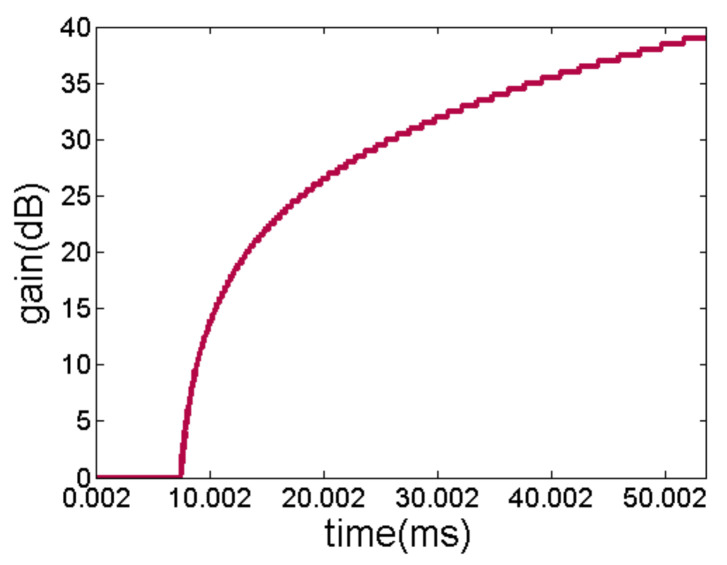
TVG configuration curve.

We compute the value of *TVG_range_* = 39 dB. We choose HMC960 as the VGA of the acquisition module. It is noteworthy that HMC960 supports the gain variation of 0 to 40 dB for two signals within the bandwidth of 100 mHz. The minimum step of gain variation is 0.5 dB, and the minimum time interval for modifying the gain is 2 μs. The dynamic TVG compensation is realized by modifying the HMC960 gain controller with SPI in FPGA. Figure 9 shows the TVG configuration curve in a positioning cycle after the acquisition module is enabled, when the *TS* of object is 10 dB.

The maximum two-way acoustic propagation attenuation *TL*_max_ corresponding to the action distance *D*_max_ is 66.96 dB. The voltage RMS *V_E_* of the input terminal of the acquisition module is expressed as follows:

(17) $$V_{E}=10^{\frac{S_{R}+SL+DI_{R}+TS-TL_{\max}}{20}}$$

when *TS* is equal to −15 dB, the resultant value of *V_E_*_min_ is 356.50 μV. Similarly, when *TS* is equal to 25 dB, the resultant value of *V_E_*_max_ is 35.65 mV. It is notable that the signal processing of the system is normal only when the analog signal RMS *V_in_* of the ADC is satisfied as presented in Equation (18):

(18) $$V_{in}=\max\left\{V_{ref}/100\sqrt{2},2^{12.5-N_{ad}}\;\times \;V_{ref}\right\}$$

**Figure 10:**
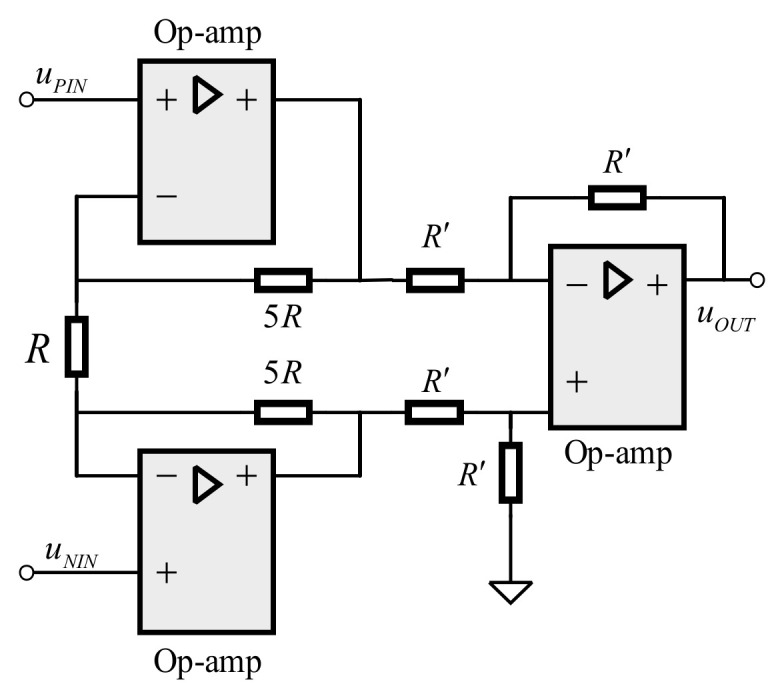
Differential instrumentation amplifier.

We calculate the amplification multiple of the conditioning circuit, the result of which is 1016. Since the conditioning circuit is composed of multi-stage analog circuits, there is an inevitable amplitude loss and because of the upper limit of gain value of HMC960 is 40 dB, thus, the first stage magnification is designed to be 11 times to meet the actual engineering requirements. As shown in Figure 10, the preamplifier circuit of the first stage uses three ADA4625 to form a differential instrument amplifier.

Here, ADA4625 is a JFET-type operational amplifier with ultra-high input impedance, the input equivalent noise is $3.3\;\mathrm{nV}/\sqrt{\mathrm{Hz}}$, and the working bandwidth is 16 mHz. The phase margin of 86° effectively prevents the self-excited oscillations.

**Figure 11:**
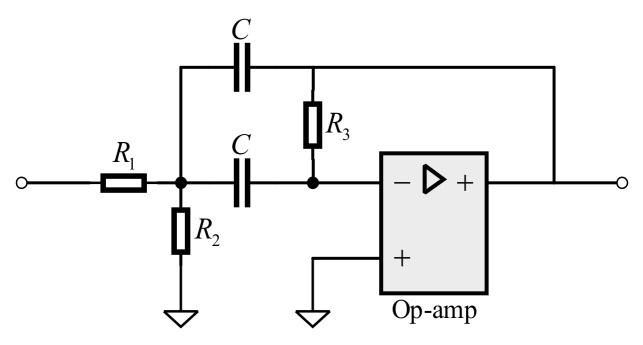
Multi-feedback band pass filter.

After the input signal of the acquisition module is amplified, the noise signal of the inclusion is also amplified. Since the system works under the narrow-band conditions, the band-pass filter is used for the second-stage signal conditioning. In order to obtain the flat passband and steep stopband, we cascade three Butterworth second-order multi-feedback (MBF) bandpass filters. This is presented in Figure 11.

**Figure 12:**
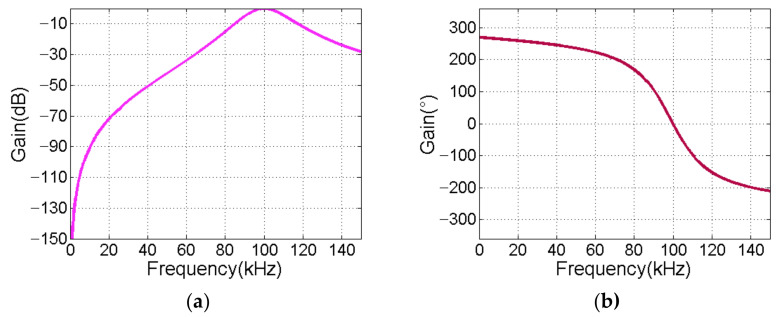
Frequency response of band pass filter. (**a**) Amplitude response; (**b**) Phase response.

Please note that the operational amplifier selects LT6233 with input equivalent noise $1.9\;\mathrm{nV}/\sqrt{\mathrm{Hz}}$. We use Equation (19) to compute *R*_1_, *R*_2_, *R*_3_ and *C*:

(19) $$\left\{\begin{array}{l} f_{0}=\sqrt{\left(R_{1}+R_{2}\right)/\left(R_{1}R_{2}R_{3}\right)}/\left(2\pi C\right)\\ A\left(jf_{0}\right)=-R_{3}/R_{1}\\ Q_{B}=\sqrt{\left(R_{1}+R_{2}\right)R_{3}/\left(R_{1}R_{2}\right)}/2 \end{array}\right.$$

where, *A*(*jf*_0_) denotes the passband gain coefficient, and *Q_B_* denotes the quality factor of the filter. Figure 12a shows the amplitude-frequency response of the cascaded bandpass filter, and Figure 12b shows the corresponding phase-frequency response. The passband bandwidth is 30 KHz. Ideally, the passband gain is 0 dB, and the phase shift at 100 KHz is 0°.

**Figure 13:**
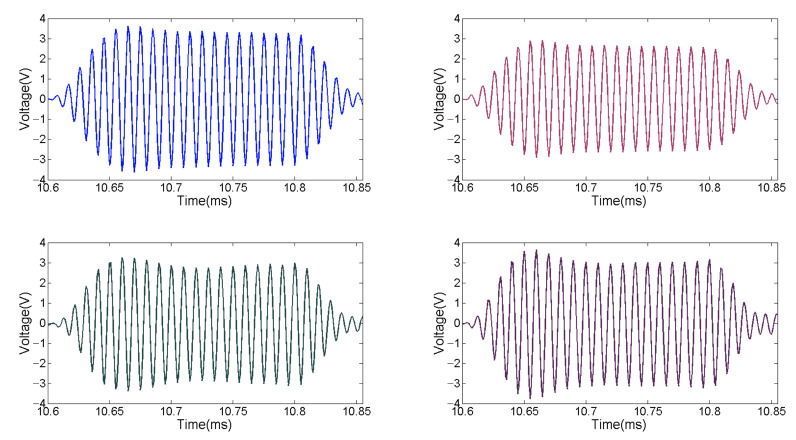
Echo signal acquisition of module #8.

According to the aforementioned analysis, the ADC selects a four-channel synchronous acquisition LTC2325-16, which integrates four parallel 16-bit SAR ADCs. The upper bound of the sampling rate is 5 MHz. It is compatible with CMOS and LVDS Serial SPI. It has a low-temperature drift internal reference voltage source of 4.096 V, and a low-power sleep mode. In the acquisition module designed in this work, the ADC works at the sampling rate of 1 MHZ. Figure 13 shows that the acquisition module #8 correctly receives the 100 KHz echo signal of the underwater target at a distance of 8 m. The number of pulses is 20 and the *TS* of object is 10 dB.

## 4. Software Design

### 4.1. Overall Software Design

**Figure 14:**
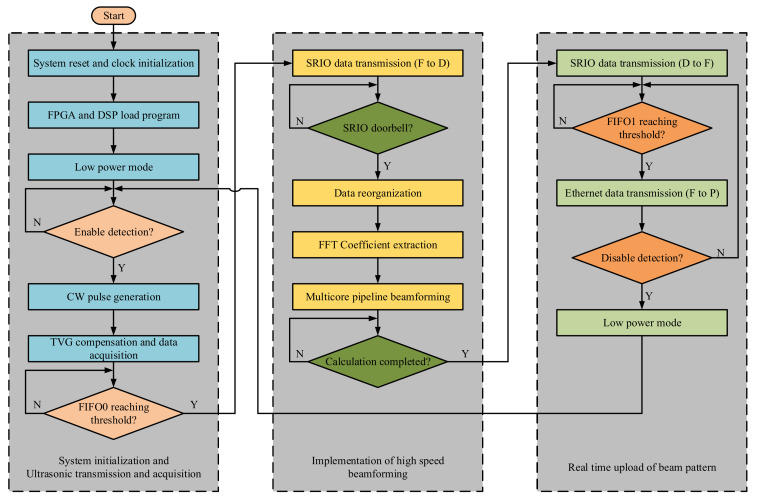
System software flow block diagram.

Figure 14 shows the programming architecture for multibeam sonar systems. First, after completing the initialization, the system enters the low-power mode. When the PC sends the startup instructions to the FPGA through the Gigabit Ethernet, the FPGA drives the transmitting module to generate CW pulse control signal, trigger the TVG compensation of each receiving channel, and control the ADC of each acquisition module for real-time data acquisition. When the amount of collected data reaches the threshold of FIFO0, the collected data is written to the memory of DSP through the SRIO interface. Then, the FPGA sends the doorbell signal to DSP after the data transmission is completed. Second, when the DSP receives the doorbell signal, the data collected in memory is repackaged. The FFT coefficients of each channel at 100 KHz are extracted (FFT in core 0 does not affect the performance of signal processor, and it is convenient for us to observe and verify the signals received by SRIO in time domain and frequency domain during software development), and 2-D CZT beamforming operation is carried out by the multi-core pipeline architecture. Finally, after the calculation of each beam pattern is completed by DSP, the beam pattern data is transmitted to FIFO1 of the FPGA through the SRIO interface. When FIFO1 caches one beam pattern, the FPGA uploads the beam pattern to the PC through the Gigabit Ethernet. In a detection cycle, the PC gets the 2-D beam pattern at each detection distance, so as to obtain the 3-D acoustic distribution of the detection sector.

**Figure 15:**
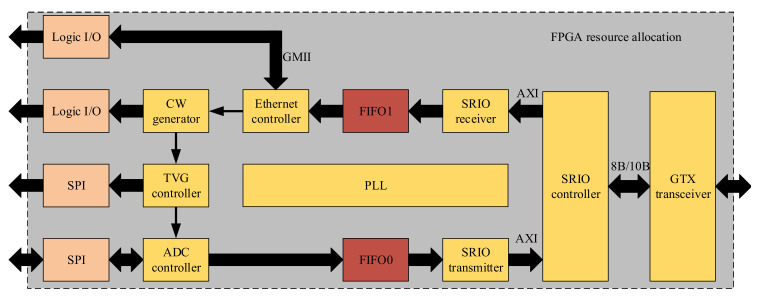
The resource allocation used in the FPGA.

Figure 15 shows the resource allocation used in the FPGA. In short, the FPGA is mainly responsible for the realization of Gigabit Ethernet communication, timing control of transmission and acquisition, generation of CW pulse logic signal, multi-channel TVG compensation, synchronous data acquisition, and management of FIFO and SRIO interfaces. It is necessary that these functions operate in a parallel state, so an XC7K325T with sufficient I/O, logic cell, and high-speed serial interface is a very suitable choice. The DSP is mainly responsible for the management of SRIO interface, the FFT coefficient extraction of data collected by each channel, the response of doorbell interrupt, and the efficient and rapid implementation of 2-D CZT beamforming algorithm.

For real-time signal processing systems, we must meet the condition that the acquisition time interval of each echo signal frame is equal to the upload time interval of each beam pattern. Therefore, in the software design of real-time systems, we must plan the data flow in the FPGA reasonably and improve the throughput of signal processing in the DSP as much as possible.

### 4.2. Analysis and Planning of Data Flow

**Figure 16:**
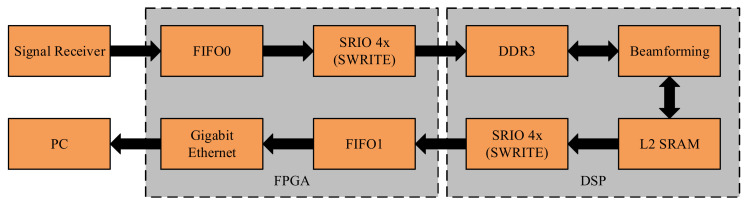
The transmission path of acquisition data.

Figure 16 shows the transmission path of acquired data in the software system. From the FPGA module to DSP module, given the time length of the echo signal frame is 256 μs, the binary data length of a single sampling channel is 16 bit, and the sampling rate is 1 Msps, the total amount of data for each echo signal frame is 32 KB. When the underwater velocity is 1500 m/s, the space distance Corresponding to a signal frame is 0.384 m, and the shortest echo receiving time interval is 512 μs. When the FPGA adopts the SRIO interface in the form of Direct I/O, the actual transmission rate of sending SWRITE package is 1795 MB/s, and the transmission of 32 KB data requires 17.408 μs. Please note that the depth of FIFO0 is greater than 32 KB. The threshold set to 32 KB enables FPGA to DSP real-time data transmission. For the data stream going from DSP module to FPGA module and then to PC, each element in the 2-D beam pattern is 16 bits in length and the number of beams is 90 × 90. The volume of a beam pattern data is 16,200 B.

**Figure 17:**
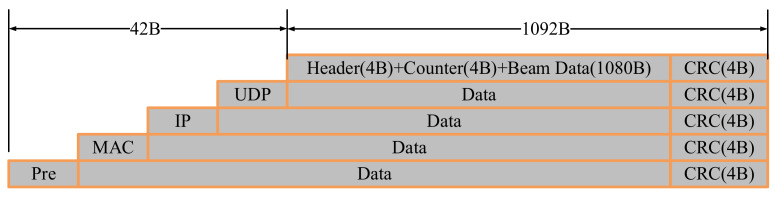
Data frame format of Gigabit Ethernet.

Figure 17 presents the Gigabit Ethernet IP message format in FPGA. A beam pattern is transmitted by using 15 IP packets. The total length of each IP packet is 1134 B. Therefore, the ideal time for Gigabit Ethernet to send 15 IP packets is 8.66 μs, so the fixed depth of FIFO1 is 2048 B, and the threshold is 1080 B. In order to achieve the purpose of real-time data transmission, the minimum time interval *T_B_* of beamforming should satisfy the expression presented in Equation (20):

(20) $$8.66\;\mu s<T_{B}<512\;\mu s$$

### 4.3. Design of 2-D CZT Beamforming Based on Multicore DSP

**Figure 18:**
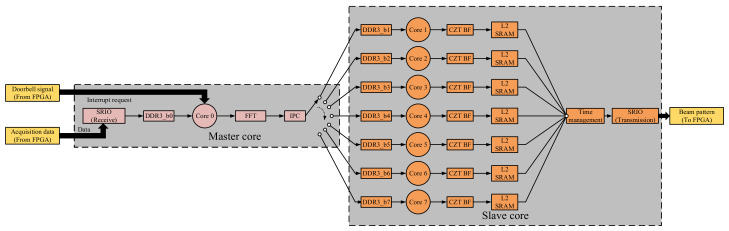
The architecture of multicore signal processing.

The TMS320C6678 has eight independent C66x cores. Each core independently accesses its own L1P, L1D, and L2 memory, and independently performs calculation using the data. However, when all the cores access DDR3 at the same time, there is an arbitration waiting period. If eight cores are used to process the echo signal data frame at the same time, the access bandwidth of DDR3 is evenly divided, consequently affecting the real-time signal processing ability of the system. In order to improve the throughput of the signal processor, we propose a 2-D CZT beamforming method based on master-slave multi-core DSP pipeline structure for the multi-beam sonar system designed in this work. Figure 18 shows the general flow of multicore signal processing (All cores will receive interrupt requests from doorbell at the same time in the eight-core parallel architecture, including the core of ongoing interrupt response, compared with the eight-core parallel architecture, the stability of the master-slave structure is higher, and the timing scheduling between cores is more rigorous).

First, the Core0 which is the main core is responsible for responding to doorbell interrupt. In the process of interrupt service, Core0 reads the acquisition data from DDR3, restores the data into a 2-D array, and converts each digital element in the array into the corresponding single-precision floating-point analog quantity.

The Core0 then performs 256-point FFT on each row of the 2-D array and modifies the amplitude and phase of the FFT coefficients of each channel at 100 KHz according to Equation (21):

(21) $$\left\{\begin{array}{l} A_{m,n}=Af_{m,n}\;\times \;Am_{m,n}\\ \varphi_{m,n}=\varphi f_{m,n}-\varphi m_{m,n} \end{array}\right.$$

where, *Af_m,n_* and *φf_m,n_* denote the amplitude and phase of the FFT coefficients of (m, n) array elements at 100 KHz. *Am_m,n_* and *φm_m,n_* represent the amplitude correction factor and phase correction value, respectively. *A_m,n_* and *φ_m,n_* represent the amplitude and phase after correction, respectively. The Core0 saves the modified FFT coefficients in an array **CS_8,8_** and stores it in DDR3. Then, Core0 sends IPC interrupt request to a sub-core. After the above operation is completed, the Core 0 exits doorbell interrupt and enters the idle state. Second, the sub-cores include Core1 to Core7. According to the CZT beamforming discussed in 2.3, in the initialization phase, each sub-core calculates the partial value of the **C***_M,N_* matrix and the 2-D FFT of **D***_L1,L2_*, and saves the calculation results in L2 SRAM. When the sub-core receives the IPC interrupt request, it enters the IPC interrupt service function. In the interrupt service function, sub-core reads matrix **CS_8,8_,** calculates **C***_M,N_*, and generates the beam intensity matrix **B***_P,Q_* according to (9). Then, the sub-core saves the **B***_P,Q_* in L2 SRAM. Finally, the sub-core uses SWRITE for transmitting **B***_P,Q_* to FPGA through SRIO interface. After the above operation is completed, the sub-core exits the doorbell interrupt and enters the idle state.

**Figure 19:**
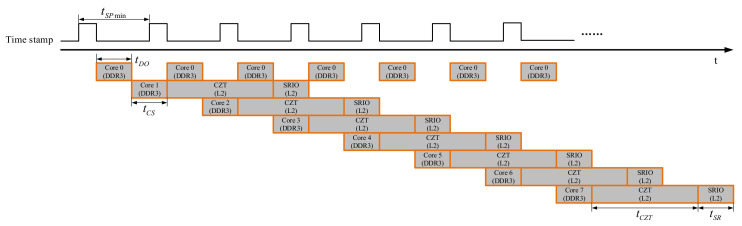
Muti-core pipeline signal processing timing.

Now, the conditions of real-time 2-D CZT beamforming in master-slave multi-core DSP pipeline architecture are further analyzed. Figure 19 shows the timing sequence of accessing memory in that architecture. The precondition of using this architecture is that any two cores are prohibited to access the DDR3 or use SRIO controller at the same time.

The real-time operating conditions of master-slave multi-core DSP pipelined signal processor is expressed as follows:

(22) $$\left\{\begin{array}{l} T_{B}=t_{DO}+t_{CS}+t_{CZT}+t_{SR}\\ 8.66\;\mu s<T_{B}\leq 7\;\times \;t_{SP\min}\\ t_{DO}<t_{SP\min} \end{array}\right.$$

where, *t_DO_* denotes the time required for Core0 to perform a doorbell interrupt service, *t_CS_* denotes the time required for the sub-core to read **CS_8,8_** from DDR3 and calculate **C***_M,N_*, *t_CZT_* denotes the time required for the sub-core to perform a 2-D CZT beamforming, *t_SR_* denotes the time required for the sub-core to transmit **B***_P,Q_* in L2 SRAM to FPGA through SRIO, and the minimum signal processing time interval of the sonar system is *t_SPmin_*. When *t_SPmin_* is 512 μs, comparing (20) with (22), the architecture increases the throughput of the signal processor by 7 times. By imposing *M* = *N* = 8 and P = Q = 90, we get L_1_ = L_2_ = 128 for the multibeam sonar system designed in this work. Table 1 shows the results of testing the time parameter *t_DO_*, *t_CS_*, *t_CZT_* and *t_SR_*. The results presented here show that the proposed sonar system has the ability to operate in real-time.

## 5. Experiment and Analysis

### 5.1. Measurment of Transmiting Sound Souce Level and Directivity

**Figure 20:**
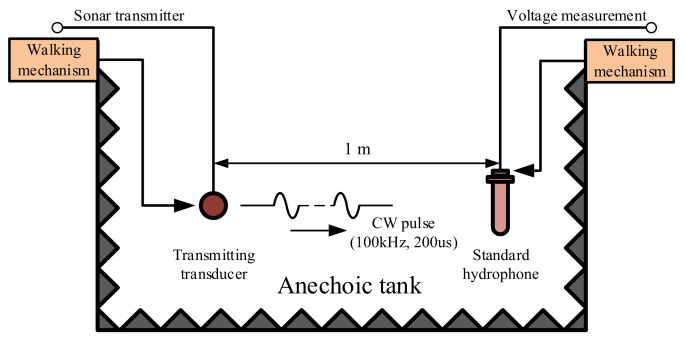
Schematic diagram of sound source level measurement.

Figure 20 shows the schematic diagram of the free field comparison method for testing the sound source level of the transmitter.

**Figure 21:**
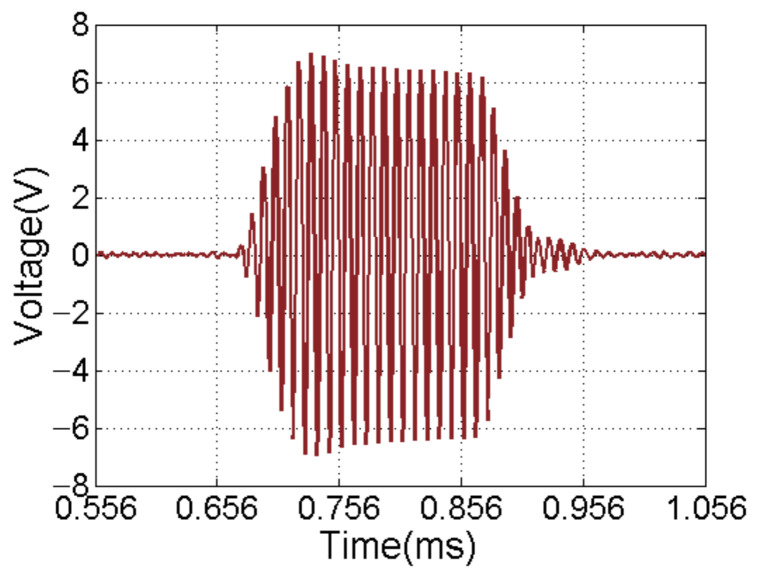
The output ultrasonic signal of standard hydrophone.

In an anechoic tank, we place the standard hydrophone at a distance of 1 m from the acoustic axis of the transmitting transducer by mechanical walking mechanism. The transmitter is used to excite the transmitting transducer to generate CW ultrasonic signals with a frequency of 100 KHz, a pulse width of 200 μs and with the repetition period of 5 s. The open circuit RMS voltage of the standard hydrophone is *U_FP_*. The calculation method of sound source level *L_SP_* is expressed as follows:

(23) $$L_{SP}=20\mathrm{lg}U_{FP}-S_{0}$$

where *S*_0_ represents the receiver sensitivity of a standard hydrophone. The value of *S_0_* for the standard hydrophone used in the experiment performed in this work is −177 dB at 100 KHz. Figure 21 shows the acoustic signal received by the standard hydrophone. The resultant value of *U_FP_* is 4.6 V. The actual sound source level of the transmitter is 190.25 dB as devised from Equation (23).

**Figure 22:**
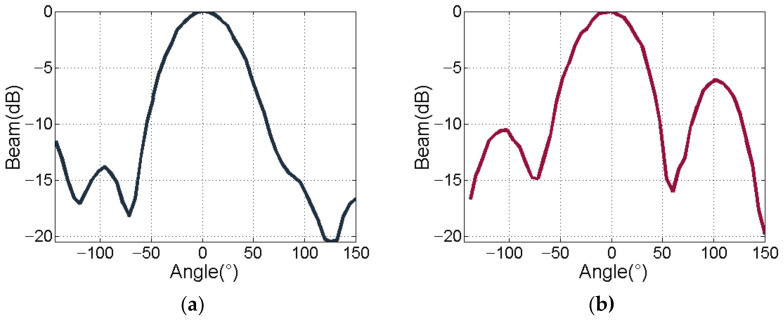
The directivity of transmitter. (**a**) Horizontal directivity; (**b**) Vertical directivity.

Now, we measure the directivity of ultrasonic emission. The standard hydrophone is placed at a distance of 1 m from the acoustic axis. First, we fix the position of the hydrophone and rotate the transmitting transducer at an angle of 2° in the horizontal direction by controlling the rotating shaft through the walking mechanism. The ultrasonic signal emitted by the transmitting transducer at each horizontal rotation angle is recorded by the hydrophone. Second, we turn the transmitting transducer over and repeat the aforementioned steps to measure the vertical directivity. Figure 22a,b shows the horizontal beam pattern and vertical beam pattern of the transmitting transducer. Please note that the horizontal −3 dB beam width is 64° and vertical −3 dB beam width is 64°. According to the above experiments, we conclude that the sound source level and beam width of the transmitter designed in this work meet the background requirements.

### 5.2. Measurement of Receiving Background Noise and Consistency

**Figure 23:**
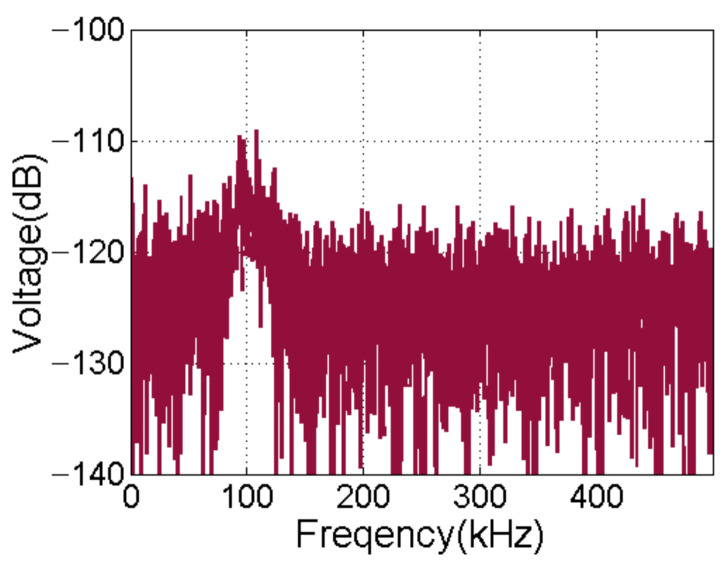
Background noise of channel #56.

The background noise and the consistency of phase and amplitude between the channels are important indexes to evaluate a multi-channel acquisition system. First, the differential instrument amplifier in the front of the acquisition channel is shorted and the TVG compensation function is disabled. Then, the sonar receiver is used for continuous acquisition of data. Figure 23 shows the frequency spectrum of the background electrical noise for channel 56. The frequency domain analysis reveals that the background noise in the passband of 100 KHz remains below −109 dB, and the noise in the stopband is very flat.

**Figure 24:**
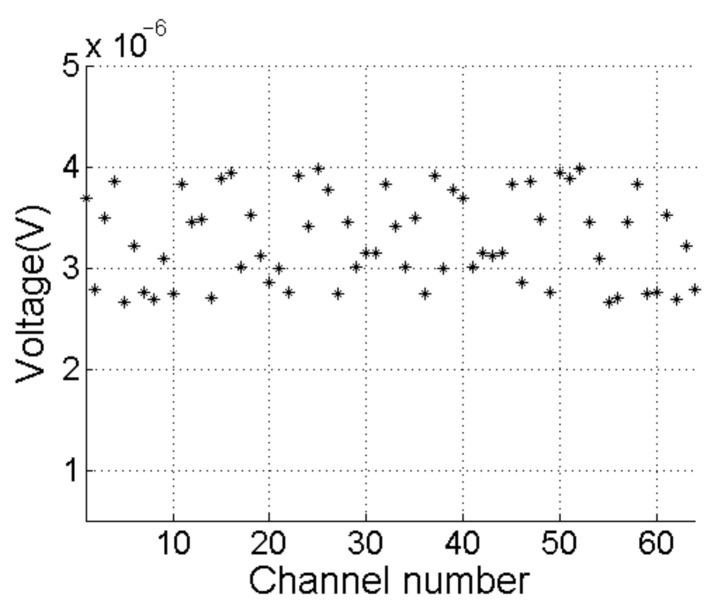
The root mean square of all channels.

Figure 24 shows the RMS value of background signals for 64 channels, which are all less than 4 μV. According to (17), at the maximum operating distance of 40 m, the minimum effective input voltage at the input of the receiver is 356.50 μV. Thus, we conclude that the background noise has little effect on performance of the proposed sonar system.

**Figure 25:**
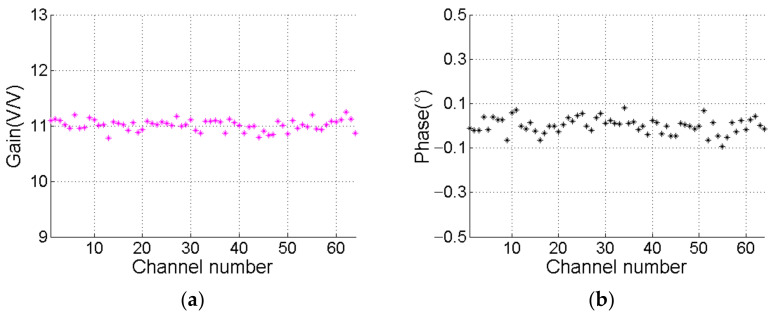
The results of consistency test (**a**) Amplitude consistency; (**b**) Phase consistency.

Second, the phase and amplitude consistency of each channel in the receiver system are the key factors that affect the positioning accuracy. We turn off the TVG compensation function of the sonar system and connect all the channels of the receiver to the same signal source. In addition, we adjust the output peak value of the signal source to 50 mV and frequency to 100 KHz continuous sine wave. Figure 25a shows the amplitude gain coefficient of each channel at 100 KHz. When channel 43 is defined as the reference channel, Figure 25b shows that the phase difference between the other channels and the reference channel is less than 0.2° and the maximum amplitude consistency error is −6.55 dB. The material error of the resistance and capacitance of the analog circuit is the major cause of the amplitude and phase error. In non-extreme working environments, the amplitude gain coefficient ratio and phase difference between each channel and the reference channel are considered to be constant. The error correction is realized by using Equation (21).

### 5.3. Underwater Positioning Experiment

**Figure 26:**
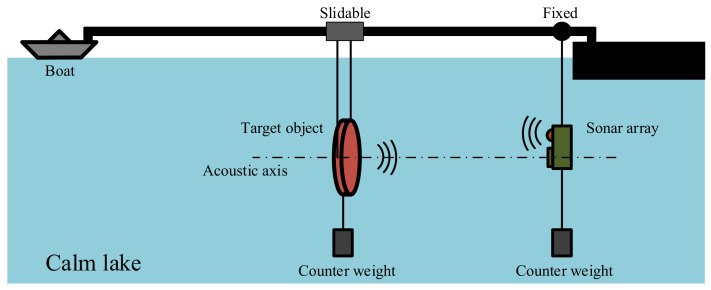
Schematic diagram of underwater positioning experiment.

Figure 26 shows the schematic diagram of the positioning experiment. The target object in the positioning experiment is a circular baffle with a *TS* of 10 dB. The baffle and the sonar array are suspended on the fixed bracket in such a way that the baffle is placed in the central acoustic axis of the receiving array. First, the baffle is placed at a distance of 5 m from the sonar array.

**Figure 27:**
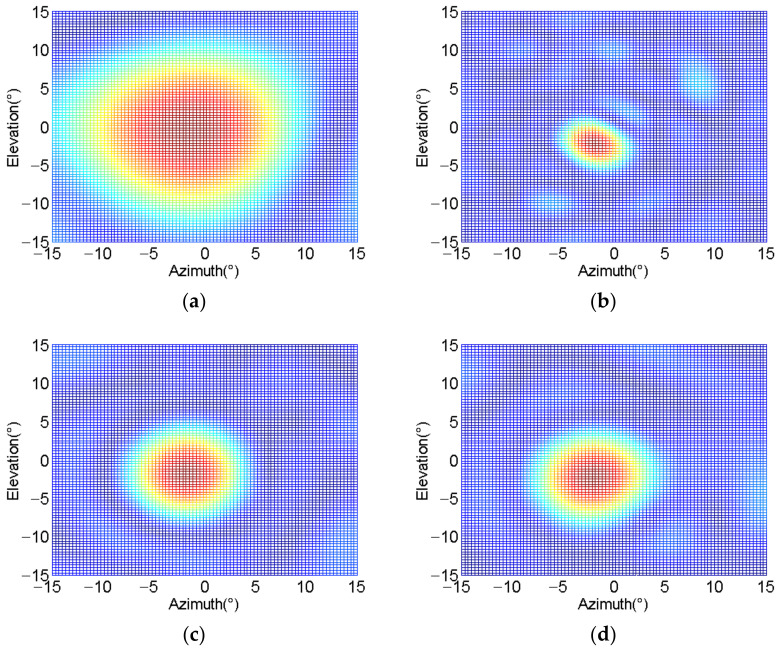
Positioning experiment. (**a**) Minimum operating distance; (**b**) Maximum operating distance; (**c**) 23 m operating distance; (**d**) 23 m operating distance.

We conduct the target positioning experiment at the minimum operating distance. Second, the baffle is placed at the distance of 40 m, and the positioning experiment is performed at the maximum operating distance. Finally, we place the baffle at a distance of 23 m from the array and the positioning experiment is carried out at the center of the detection sector. The actual sound speed used in the experiments performed in this work is 1481.6 m/s. The transmitted signal is set to a CW sine wave with a frequency of 100 KHz and a pulse width of 200 μs with a repetition period of 54 ms. The multi-beam sonar system produces the number of 94 beam patterns in a detection period. The host computer receives the beam pattern uploaded by the multi-beam sonar system in real-time through Gigabit Ethernet. Figure 27a shows that beam pattern 0 locates the position of the baffle at the minimum action distance. In addition, it is estimated that the central position of the baffle is in −1.0° in horizontal direction and 0.7° in vertical direction. Figure 27b shows that beam pattern 93 locates the position of the baffle at the maximum operating distance, which is located at −2° in horizontal direction and −2.3° in vertical direction. In many positioning experiments with a working distance of 23 m, we observe that the beam pattern that can be positioned to the baffle is evenly distributed in 46 and 47. Figure 27c,d present the results of different times during the positioning experiment of 23 m working distance. However, please note that both of them locate the baffle at the position of −1.6° in the horizontal direction and −1.3° in the vertical direction. The main reason for the positioning angle deviation is that it is difficult to achieve accurate physical alignment between the center position of the baffle and the hydrophone array. The uneven roughness of the baffle surface causes the scattering of the acoustic wave. In addition, the influence of the non-stationary characteristics of the underwater acoustic channel on the acoustic phase also plays its role. On the basis of the aforementioned experiments, it is evident that the proposed multi-beam sonar system has the ability to realize 3-D positioning within the range of 5 m to 40 m in the application of underwater detection.

### 5.4. Discussion on Scalability of Multibeam Sonar

Usually, generating the size of 90 × 90 beams in detection sector is enough for satisfying most of the underwater positioning requirements. According to the related description presented in *2.2*, the size of CZT beamforming is 128 × 128 and the elements of the planar array is up to 39 × 39. According to Figure 8, the number of I/O consumed by one acquisition module is 11 and the maximum number of FPGA users I/O is 500. The receiver uses *Q_A_* acquisition module for data acquisition and one FPGA chip for signal aggregation and communication. When the number of I/O in one FPGA chip is not enough, *Q_F_* FPGA chip is added. The echo signal frame is represented by 256 sampling points. The size of an echo signal frame received by the receiver is 2*Q_A_* KB. The maximum input data rate is 3.82*Q_A_* MB/s, and the communication rate between FPGA and DSP is 1795 MB/s. The average time consumed by DSP for data reorganization of a channel and performing 256-point FFT is 3.07 μs. The working time of each sub-core is 3009.78 μs. According to Equation (24), the relationship between *Q_A_* and *Q_F_* is expressed as:

(24) $$\left\{\begin{array}{l} Q_{A}<45.45\left(Q_{F}+1\right)\\ Q_{A}\leq 381\\ 4Q_{A}<\left(7T_{p}-3009.78\right)/3.07 \end{array}\right.$$

where, *T_p_* denotes the minimum signal processing time interval. The *T_p_* of the multi-beam sonar system proposed in this work is 512 μs, the maximum value of *Q_A_* is 45 and the corresponding *Q_F_* is 0. This is extendable to at least 144 receiving channels and a receiving array of 12 × 12.

## 6. Conclusions

In this work, we comprehensively present a design method of real-time multi-beam sonar system with the characteristics of 90 × 90 beams, 0.384 m distance resolution, 190.25 dB sound source level, and background noise less than 4 μVrms to realize real-time 3-D position of target object in 30° × 30° sector at a distance of 5 m to 40 m. The contradiction between real-time positioning and multi-channel signal processing in multi beam sonar system is solved under the condition presented in (22). In the algorithm research, three beamforming algorithms, i.e., D&S, DM, CZT, are studied. According to the design background, the CZT beamforming algorithm has the highest efficiency and is adopted in this work. In terms of hardware design, the design method of a high frequency CW pulse transmitting module with 190.25 dB sound source level and an acquisition module with 40 dB dynamic range and noise less than 4 μVrms are presented. In terms of software design, the data transmission path between FPGA and DSP as well as the host computer is quantitatively analyzed. Based on SRIO and Gigabit Ethernet, the data transmission path is planned, and the real-time data transmission between each module is realized. The novel signal processor design method of master-slave multi-core pipelined architecture is presented. In the proposed architecture, the throughput of signal processor is seven times higher than that of single-core operation. Consequently, the time complexity of CZT beamforming is reduced rapidly. Finally, the underwater positioning experiment proves that the multi-beam sonar system designed in this work has the ability to realize the real-time 3-D positioning with the performance of maximum deviation of azimuth is 2°, the maximum deviation of elevation is 2.3°, and the maximum distance deviation is 0.379 m.

## Figures and Tables

**Table 1:** The testing of time parameter.

$t_{SP\min}$	$t_{DO}$	$t_{CS}$	$t_{CZT}$	$t_{SR}$
512 μs	190.45 μs	6.32 μs	2994.26 μs	9.2 μs

## Data Availability

Not applicable.
